# Acute rhabdomyolysis in a young woman with moderate COVID-19

**DOI:** 10.1016/j.idcr.2021.e01212

**Published:** 2021-06-29

**Authors:** Kohei Fujita, Osamu Kanai, Kazutaka Nanba, Naoki Esaka, Hiroaki Hata, Koichi Seta, Takao Odagaki

**Affiliations:** a Department of Infectious Diseases, National Hospital Organization Kyoto Medical Center, Kyoto, Japan; b Division of Respiratory Medicine, Center for Respiratory Diseases, National Hospital Organization Kyoto Medical Center, Kyoto, Japan; c Department of Endocrinology and Metabolism, National Hospital Organization Kyoto Medical Center, Kyoto, Japan; d Department of Gastroenterology, National Hospital Organization Kyoto Medical Center, Kyoto, Japan; e Department of Surgery, National Hospital Organization Kyoto Medical Center, Kyoto, Japan; f Department of Nephrology, National Hospital Organization Kyoto Medical Center, Kyoto, Japan

**Keywords:** COVID-19, SARS-CoV-2, Rhabdomyolysis, Muscle training, Exercise

## Abstract

The coronavirus disease 2019 (COVID-19) pandemic caused by severe acute respiratory syndrome coronavirus 2 (SARS-CoV-2) is having serious medical, social, and economic impacts worldwide. COVID-19 may lead to a variety of complications, including rhabdomyolysis. Although rhabdomyolysis is a rare complication, it can lead to severe kidney damage. Recent studies suggest that rhabdomyolysis caused by SARS-CoV-2 is more common in middle-aged and older men with severe COVID-19. Herein we report a case of rhabdomyolysis in a young woman with moderate COVID-19. She had a habit of muscle training. She presented with moderate COVID-19 and acute rhabdomyolysis that required a large volume of fluid infusion in addition to dexamethasone and remdesivir. Clinicians should pay attention to the development of rhabdomyolysis in patients with COVID-19, especially those with a habit of strenuous exercise or muscle training, even if they are young and have moderate COVID-19.

## Highlights

• COVID-19-associated rhabdomyolysis is most common in older men with severe disease.
• This case of rhabdomyolysis occurred in a young woman with moderate COVID-19.
• She practiced daily strength training, which may have increased her risk.
• She was treated with intravenous fluid infusion, dexamethasone, and remdesivir.

## Introduction

Rhabdomyolysis is a syndrome characterized by muscle necrosis and the release of intracellular muscle constituents into the systemic circulation. The most common complication is acute kidney injury (AKI). A prompt diagnosis is essential for successful treatment. Although trauma due to accidents or disasters is a well-known cause of rhabdomyolysis, drug-induced adverse reactions and infections can also be triggers.

Creatine kinase (CK) is the most sensitive indicator of muscle damage [1]. Rhabdomyolysis should be suspected if CK levels are above 5000 U/L [2]. Early fluid rehydration is the most important measure for the prevention of AKI [2].

Various viral infections such as influenza A and B, herpes simplex, adenovirus, echovirus, human immunodeficiency virus, and cytomegalovirus, can cause rhabdomyolysis [3,4]. Recently, severe acute respiratory syndrome coronavirus 2 (SARS-CoV-2), which causes coronavirus disease 2019 (COVID-19), has also been reported as a cause of rhabdomyolysis [[5], [6], [7]]. Previous reports have shown that rhabdomyolysis in COVID-19 patients typically occurs in middle-aged men and those with severe COVID-19 [[5], [6], [7]]. The present case differs from ones in these previous reports in that she was a young woman with moderate COVID-19. Current knowledge of the complications of COVID-19 is limited, and so the accumulation of case reports is important to establish optimal management of COVID-19 patients. Here, we present a rare case of acute rhabdomyolysis in a young woman with moderate COVID-19.

## Case presentation

**Fig. 1:**
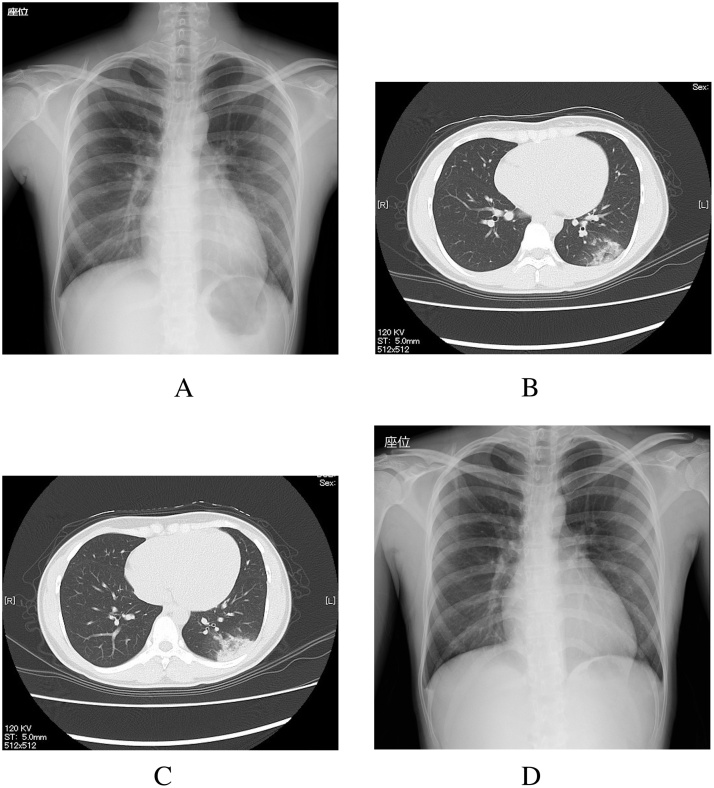
Chest imaging showing signs of COVID-19. A: Chest X-ray showing an abnormal shadow in the left lower zone on admission. B, C: Chest computed tomography showing a patchy infiltrative shadow in the left lower lobe. D: Chest X-ray showing improvement of the abnormal shadow on the day 8.

**Fig. 2:**
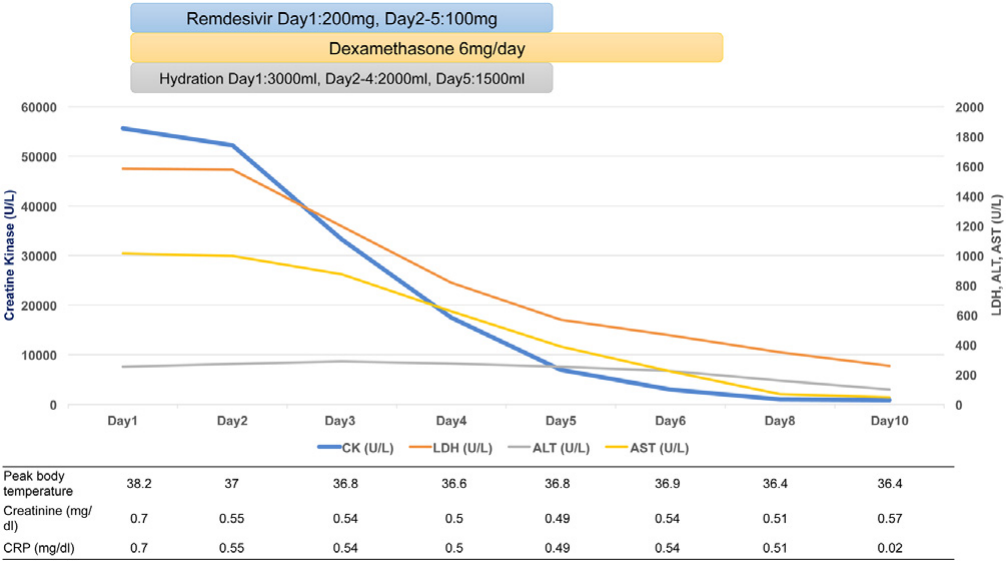
Clinical course and treatment of COVID-19 and rhabdomyolysis.

A 19-year-old woman was admitted to our hospital with dry cough, high fever (≥38℃), and fatigue lasting 3 days. She also had difficulty raising her arms due to muscle pain. Chest X-ray showed an abnormal shadow in the left lower lung zone (Fig. 1A). Computed tomography revealed a patchy infiltrative shadow in the left lower lobe (Fig. 1B, C). She was tested for SARS-CoV-2 using a polymerase chain reaction test, which was positive, confirming the diagnosis of COVID-19. She was living in a dormitory of police training academy, but none of her roommates were infected. Her parents and siblings, whom she saw on weekends, were also not infected. Unfortunately, the route of infection was unknown. Blood biochemistry on admission revealed markedly elevated levels of serum CK (55,613 U/L), lactate dehydrogenase (1,583 U/L), myoglobin (3,031 ng/mL), aspartate aminotransferase (1,013 U/L), and alanine aminotransferase (252 U/L). Her urinary myoglobin level (9,070 ng/mL) was also high, but her renal function remained normal. There were no abnormalities in her thyroid function. Her anti-nuclear, anti-RNP, anti-SM, anti-Scl-70, anti-Jo-1, and ds DNA antibodies were all negative, ruling out a diagnosis of autoimmune myositis. She was a student of police training academy, and as part of training, she routinely practiced daily muscle training. She had been doing 50 push-ups, 50 sit-ups, and 50 squats every morning and evening as part of her training. Treatment for both COVID-19 and rhabdomyolysis was initiated after admission. The clinical course is shown in Fig. 2. For COVID-19 treatment, she received a combination of dexamethasone for 7 days and remdesivir for 5 days. A chest X-ray on day 8 confirmed improvement of her pneumonia (Fig. 1D). Her respiratory symptoms also improved. For rhabdomyolysis treatment, she received a five-day continuous infusion of a large volume of fluid to prevent AKI. She was able to establish sufficient urine flow during transfusion and maintain normal renal function. Her serum CK levels dropped from 55,613 U/L on day 1 to 856 U/L on day 10. Her illness improved, and she was discharged on day 11.

## Discussion

The COVID-19 pandemic has impacted human health as well as global economy. COVID-19 causes not only pneumonia, but has various other manifestations, such as neurologic manifestations [8], renal dysfunction [9], and cardiovascular implications [10]. Several recent studies have revealed that SARS-CoV-2 infection can cause rhabdomyolysis concomitant with COVID-19. Most case reports of COVID-19-associated rhabdomyolysis have been of middle-aged men with severe COVID-19 [[5], [6], [7]]. In the present case, COVID-19 was moderate, but the patient’s serum CK level was abnormally high. There was a risk of developing AKI within a few days of hospitalization. Although she had a habit of muscle training, the abnormally high elevation of serum CK levels could not be explained by that alone. In accordance with a previous study evaluating serum CK levels in exertional muscle damage, the average peak CK levels were 6420 U/L, 2100 U/L on day4 and 7 after the exercise [11]. The CK levels of our patient cannot be explained by exercise alone. She had no history of medication or disease that could cause rhabdomyolysis. We believe that it is natural to assume that the SARS-CoV-2 caused the rhabdomyolysis. Thus, we speculate that SARS-CoV-2 infection strongly contributed to the development of rhabdomyolysis. The rhabdomyolysis may have occurred as a result of daily overuse of her muscles combined with SARS-CoV-2 infection. The mechanism by which viral infections cause rhabdomyolysis may include direct viral invasion to muscles [12,13], toxin-induced muscle damage, or secondary damage due to an immune response against viruses [3]. Direct viral invasion of the muscle has been described with influenza virus [12] and herpes virus [13], but has not been described with SARS-CoV-2. Although angiotensin-converting enzyme 2, which is the functional receptor for both severe acute respiratory syndrome coronavirus (SARS-CoV) and SARS-CoV-2, is known to be present in skeletal muscle [14], SARS-CoV was not detected in skeletal muscle on histological analysis in one study [15]. SARS-CoV-2 may not be present in skeletal muscle in cases of COVID-19-associated rhabdomyolysis. Because cytokine storm is considered to be the main pathogenetic factor in determining the severity of COVID-19 [16], secondary damage due to immune response may be the main cause of rhabdomyolysis in patients with COVID-19. Furthermore, even if COVID-19 is moderate as in the present case, it is likely that rhabdomyolysis can occur if the muscles are exhausted and vulnerable. In contrast to the present case which showed acute onset, one previously reported case showed late onset in a patient with long COVID syndrome [6]. In patients with COVID-19, rhabdomyolysis can occur acutely or chronically. SARS-CoV-2 is an emerging virus, and COVID-19 is an emerging infectious disease, and much is still not well understood. The accumulation and analysis of similar case reports is important to gain a better understanding of COVID-associated rhabdomyolysis.

In conclusion, we observed acute rhabdomyolysis in a young woman with moderate COVID-19. Clinicians should pay attention to the development of rhabdomyolysis in patients with COVID-19, especially those with a habit of strenuous exercise (aerobic exercise such as swimming, jogging, cycling, etc.) or muscle training (anaerobic exercise, as represented by strength training, weightlifting, etc.).

## Funding

This research did not receive any specific grant from funding agencies in the public, commercial, or not-for-profit sectors.

## Ethical approval

Our institution does not require IRB approval for case reports.

## Consent

We obtained written consent form from the patient and her family.

## Author contributions

KF drafted and revised the manuscript.

KF, OK, KN, NE, HH, KS, and TO treated the patient and reviewed the manuscript.

All authors approved the manuscript for submission.

## Declaration of Competing Interest

The authors report no declarations of interest.
